# The Impact of the COVID-19 Pandemic on Dental Education: An Online Survey of Students’ Perceptions and Attitudes

**DOI:** 10.3390/dj9100116

**Published:** 2021-10-09

**Authors:** Ana Badovinac, Matej Par, Laura Plančak, Marcela Daria Balić, Domagoj Vražić, Darko Božić, Larisa Musić

**Affiliations:** 1 Department of Periodontology, School of Dental Medicine, University of Zagreb, HR-10000 Zagreb, Croatia; badovinac@sfzg.hr (A.B.); vrazic@sfzg.hr (D.V.); bozic@sfzg.hr (D.B.); 2 Department of Endodontics and Restorative Dentistry, School of Dental Medicine, University of Zagreb, HR-10000 Zagreb, Croatia; mpar@sfzg.hr; 3 School of Dental Medicine, University of Zagreb, HR-10000 Zagreb, Croatia; lplancak@sfzg.hr; 4 Private Dental Practice, HR-10000 Zagreb, Croatia; balic.marcela@gmail.com

**Keywords:** dental education, dental students, online learning, COVID-19, surveys and questionnaires

## Abstract

Purpose: Dental education institutions worldwide experienced disruptive changes amid the COVID-19 pandemic, with a rapid switch to the online learning format. Thus, this study aimed to assess the impact of the COVID-19 pandemic on dental education and evaluates the perceptions and attitudes of students towards the introduction of online learning in the School of Dental Medicine in Zagreb, Croatia. Methods: A survey was conducted on a population of undergraduate students. It was comprised of perceptions and attitudes of students on the impact of the COVID-19 pandemic on their psychoemotional status, changes introduced in the educational system, and online learning in particular. Results: Of the 352 students that completed the survey, 66.2% of students reported being psychoemotionally affected by the lockdown. The most significant impact of the switch from in-person to online learning was observed in terms of missing contact with lecturers (60.3%) and peers (90.3%) and loss of practical courses, regarding which 65% of students agreed that they could not be compensated. While only 36.1% reported that online teaching fully met their expectations, the majority of the students (61.9%) agreed that online lectures were as valuable as in-person lectures and that the theoretical courses could be carried out online in the future as well (69.9%). Conclusions: Students reported relative satisfaction with changes in the learning format and a positive attitude towards online learning; however, several challenges and obstacles were identified.

## 1. Introduction

After less than three months of the first official report on the identification of the novel coronavirus in China, now known as SARS-CoV-2, the World Health Organisation declared a global pandemic in mid-March 2020 due to its unprecedented spread rate [1,2,3]. Exposure to infectious respiratory fluids was defined as the main mode of viral transmission, mostly in a non-contact manner through droplets and aerosols [4,5,6,7]. A series of behavioural and social measures, among which physical distancing, was recommended and introduced to prevent and reduce viral transmission [8,9].

Educational institutions worldwide responded by cessation of education onsite and introduction of remote learning. United Nations Educational, Scientific and Cultural Organization (UNESCO) reported that at the peak of the crisis in March and April 2020, more than 1.6 billion learners of 190 countries were affected by school closures, and some still remain affected [10]. In Croatia, all onsite university-level classes were suspended on 13 March 2020 and transferred to online learning until the end of the academic semester.

Given that the dental curriculum features theoretical education and practical training, switching to online learning, which is generally applicable to the theoretical content, greatly affected dental faculties worldwide. Early released position papers addressed the challenges dental education faced in the wake of the pandemic: the infrastructure, no access to preclinical and clinical training, and meeting the examination and graduation requirements [11,12,13,14].

Online learning is certainly not a novelty. In fact, higher education institutions initially started with online courses as early as the beginning- and mid-1990s [15]. With the development of technology, online learning has become more accessible and diverse. Synchronous online learning runs similar to traditional classes, as the lecturer and students are present in the same interactive online environment at different physical locations. Examples of this mode of online learning are online lectures/webinars, virtual congresses, or real-time chats. Asynchronous learning modes allow the students to interact with the educational material and the teacher on their own flexible schedule. Examples of asynchronous learning are pre-recorded video lessons, lectures and viewing demonstrations, reading sources and even virtual libraries, and research projects. Both modes may use features such as audio, video, text, or even interactive apps and blackboards [16].

The undergraduate study programme at the School of Dental Medicine University of Zagreb spans over 12 semesters; the first to sixth semesters include basic medical and theoretical dental and preclinical courses, while clinical courses are introduced from the seventh semester onwards. Before the COVID-19 pandemic, the teaching of all course forms was primarily delivered in person. However, the digitalisation of teaching and examination has long been introduced through a remote learning system Merlin, provided by the Croatian Academic and Research Network (CARNet). With the cessation of in-person teaching in March 2020, synchronous (Zoom Cloud Meetings, Google Meet, Skype) and asynchronous (Merlin) formats were exclusively used to deliver online lessons until the end of the academic semester. The examination and assessment of knowledge were also conducted remotely, using synchronous formats. As the epidemiological situation improved, certain examinations were conducted in person under particular conditions (use of personal protective equipment (PPE), maintenance of physical distance, maximum 2 students).

In 2021 we are witnessing a gradual return of in-person educational activities. However, due to the unpredictability of the contagion and subsequent public health policies, the development of flexible curricula inclusive of online learning is adamant. Wagner et al. [17] highlighted that the success of online learning lies in the rate to which it meets the needs and addresses the concerns of the key stakeholders. Students, in fact, represent one of the stakeholder groups as they are the direct consumers of online learning. Creating an effective framework for it requires identifying barriers and enablers/solutions [18,19].

The aim of this study was to assess the impact of the COVID-19 pandemic on dental education and evaluate the perceptions and attitudes of students towards the introduced online learning in the School of Dental Medicine in Zagreb, Croatia.

## 2. Materials and Methods

### 2.1. Study Design

This is a cross-sectional observational study that was conducted using an electronically distributed survey on a population of undergraduate students enrolled in the academic year 2019/2020.

### 2.2. Ethical Considerations

The study was approved by the Ethics Committee of the School of Dental Medicine (No: 05-PA-30-XIX-9/2020). Participant information and a consent form were provided in written form before the beginning of the survey. Participation was voluntary and anonymous, and the participants could withdraw at any point.

### 2.3. Survey

A survey was developed for the purpose of this study. It was prepared in Google Forms and sent via e-mail to all of the undergraduate students enrolled in the academic year 2019/2020. The survey was conducted in September 2020, after the official end of the summer exam session. It was open for a total of two weeks, and a reminder was sent after four and ten days. As it was stated in the participant information, the statements referred to the period of the switch from in-person to remote learning during Croatia’s lockdown and until the end of the academic semester and exam session.

The survey was created de novo for the purpose of this study by a group of four researchers with previous experience in dental education research, one postgraduate student, and one undergraduate student. The development commenced with a focus group discussion. Next, a pool of preliminary statements was made based on the main research question. The questions were further refined, divided into thematic sections, and the final number was reached upon mutual agreement. The first section on sociodemographic data included information regarding gender, age, year of study, place of residence, and living circumstances during the “lockdown” period. The second section of three statements focused on the self-perceived psychological impact of the SARS-CoV-2 contagion and the introduction of strict public health policies and measures. The third section consisted of 15 statements on attitudes and opinions about changes introduced to learning and teaching during the pandemic and loss of clinical practice in the summer semester 2019/20. Finally, the fourth part was assessed through 15 statements attitudes and perceptions of online learning and teaching. Participants evaluated their agreement with the statements on a five-point Likert type scale, ranging from 1 (strongly disagree) to 5 (strongly agree).

### 2.4. Data Analysis

Responses “completely disagree” and “disagree” were regarded as disagreement with the statement, while “agree” and “completely agree” as an agreement. The statement “neither agree nor disagree” was regarded as a neutral response. Categorical data were reported as frequencies and percentages, whereas the data for the Likert type scale were represented by mean ± SD. Differences in responses to statements S1–S33 between genders, as well as between the groups of preclinical and clinical students, were tested using a two-tailed *t*-test for independent observations and homogeneous variance [20]. An explanatory factor analysis using principal component analysis with varimax rotation was performed to investigate underlying factors explaining students’ responses to S1–S33 [21].

The statistical analysis was performed using SPSS (version 25, IBM, Armonk, NY, USA) at an overall level of significance of α = 0.05.

## 3. Results

### 3.1. Demographic Data

The demographic characteristics of the participating students are presented in Table 1. Of the 568 students enrolled in the academic year 2019/20 at the School of Dental Medicine University of Zagreb, 352 (62.0%) participated and completed the survey. The mean age was 22.5 ± 2.0 years, and 83.5% of the participants were female. According to their year of study, the proportion of respondents (%) was not equally distributed; however, relatively similar, with most respondents in their fifth year (20.2%) and least in the first year (13.1%) of study. Three hundred and eleven (88.4%) students lived with their families and 323 (91.7%) in their place of residence during the lockdown. Twenty-seven (7.7%) students were tested for SARS-CoV-2, of which six (1.7%) tested positive. Five out of six students who tested positive reported experiencing prejudice due to SARS-CoV-2 infection.

### 3.2. The Self-Perceived Psychoemotional Impact of the COVID-19 Pandemic

Data on the psychoemotional impact of the COVID-19 pandemic is shown in Table 2. Students reported different perceptions of the viral emergence and subsequent possibility of infection versus an introduction of a radical public health measure, i.e., lockdown. Around one-third (31.3%) of students reported feeling worried and insecure about the emergence of the SARS-CoV-2, while 26.4% expressed fear about the possible infection. However, two-thirds of the students (66.2%) reported that the lockdown was the cause of feelings of anxiety and insecurity. No differences between preclinical and clinical students were observed. Female students, however, expressed more significant concern and insecurity with regards to the introduction of lockdown (*p* = 0.043).

### 3.3. The Self-Perceived Educational Impact of the COVID-19 Pandemic

Data on the impact of the COVID-19 pandemic on education and educational changes are presented in Table 3. The majority of the students (62.8%) expressed concern about the outcome of the academic year 2019/20, female students significantly more so (*p* < 0.001). In general, it seems that the COVID-19 pandemic did not necessarily influence students’ perception of the academic year as more stressful, their motivation for studying, and their perception of studying as more difficult; however, significant differences in the responses could be observed among female students and preclinical students. Female students had significantly more agreed (*p* < 0.001) that the semester was more stressful than the previous ones, and both females and preclinical students agreed more that they were not equally motivated to study (*p* = 0.038 and *p* = 0.014, respectively) and that studying was more challenging during the COVID-19 pandemic (*p* = 0.005 and *p* = 0.002, respectively). Almost half of the respondents (47.1%) agreed that they had more time to study, which they have used well and have benefited from.

As many as 90.3% of the students agreed that they had missed the social contact with colleagues. A total of 60.3% of the students missed the social contact with teachers, and preclinical students were more likely to agree (*p* = 0.001). Half of the participants reported that the teachers were available for contact and communication as usual.

The students did not seem to be particularly negatively affected by the adaptation in knowledge assessments. However, online exams and exams in presence with the use of PPE seemed to have been a more significant source of stress for female students (*p* = 0.034 and *p* = 0.05, respectively).

Students expressed significant concerns about the loss of practical courses. A total of 65.0% of students disagreed that the lost practical courses can be fully compensated. Clinical students were significantly more likely to disagree with that statement (*p* < 0.001). Furthermore, almost half of the students, 47.1%, reported that due to this loss, it was more challenging to adopt the learning material needed for the knowledge assessments, and has, thus, affected their knowledge during the examination (45.7%).

When students were asked about the opportunities to compensate for the lost practical courses during summer and/or winter holidays, 58.8% of students agreed with this possibility, with clinical students agreeing significantly more (*p* < 0.001). On the contrary, if they were offered to compensate for the suspended practical courses by extending their study programme, only 25.6% of the students would agree to it. Preclinical students were significantly more likely to disagree with that opportunity (*p* < 0.001).

### 3.4. Students’ Attitudes and Perception of Online Learning during the COVID-19 Pandemic

Data on the perception and attitudes towards online learning is presented in Table 4. The majority of the students (91.8%) agreed that they had all the prerequisites to follow online classes. However, 37.5% reported that they had encountered technical issues that made it impossible to follow online classes. More than half of the students (58.5%) agreed that the teaching faculty managed to organise online classes well in the short time frame, and 61.3% stated that the quality of online classes improved over time. High levels of agreement were recorded in the statements that the quality of online classes differed among subjects and among the members of the teaching faculty, 88.9% and 95.5%, respectively.

Around one-third (35.3%) of the students agreed that they had received the same amount of information during online classes as did the previous generations in the same period. Female students had a significantly higher level of disagreement with that statement (*p* = 0.039). Nearly two-thirds (64.5%) reported that online teaching influenced the amount of the acquired knowledge, while more than half of the respondents (52.6%) believed that online teaching influenced the results of the exams they sat.

The majority of the students (61.9%) agreed that online lectures were as valuable as in-person lectures, while 69.9% of the students agreed that lectures and theoretical courses could be carried out online in the future as well. A total of 34.3% agreed that they would master the curriculum more successfully if in-person. More than half of the students (57.7%) stated that they were able to focus more during online lectures than they would during onsite lectures.

Finally, around one-third of the respondents (36.1%) agreed that online teaching fully met their expectations, while 44.4% agreed that the use of online education platforms prepared them well for further education and professional improvement.

### 3.5. Attitudes toward SARS-CoV-2, Teaching and Online Teaching during COVID-19 Pandemic Grouped in Dimensions by Explanatory Factor Analysis

The factor analysis identified nine principal components with eigenvalues ≥1. Of these, the first four principal components were selected as the main factors that explained the responses to S1–S33. These four principal components explained cumulatively 43.9% of the total variance. The variance percentages explained by individual principal components (1st–4th) amounted to 20.9, 9.6, 7.1, and 6.3, respectively.

The loadings of statements S1–S33 with regards to their respective principal components are presented in Table 5. The interpretation of factors and the number of statements grouped within each factor (principal component) is as follows:

1. Modality of teaching—seven statements related to various aspects of offline and online schooling;
2. Impact of distress—five statements related to learning abilities in the period of the pandemic;
3. Satisfaction with online teaching—five statements related to online teaching;
4. Response to the pandemic—four statements related to the impact of the pandemic.

## 4. Discussion

The present study evaluated the self-perceived impact of the COVID-19 pandemic on dental education and students’ perspectives and attitudes towards online learning. The obtained data suggest a generally positive perception of online learning and its continued use in the future; however, several obstacles to a better satisfaction rate were identified.

Several published studies evaluated the psychological impact of COVID-19 on the population of students. An Italian survey of 501 university students from Rome reported that the COVID-19 pandemic put the student population at risk for psychological distress, and one of the factors associated with the increase of anxiety was the female gender [22]. A large study on 2534 university students in the United States highlighted that the majority of the respondents, 45%, experienced a high psychological impact from COVID-19 [23]. Furthermore, a Turkish study on a population of dental students reported higher anxiety scores in females and clinical students [24]. The data obtained with this present study, while limited, suggests that the most significant source of feelings of fear and insecurity in students was the introduction of a very radical public health policy/measure, i.e., the lockdown. The emergence of the novel virus and contagion with it caused distress to a lesser extent. While there is no clear explanation for this finding, it could be speculated that the lockdown represents a novel situation that is characterised by what can be evaluated as potential stressors: coping with fears and insecurity and isolation [25,26,27].

Interestingly, one of the identified factors (“Response to the pandemic”) suggests that the students who most frequently reported feelings of insecurity due to the virus emergence, fear of contagion, and worriedness due to the lockdown also reported worry about the academic year outcome. Another of the identified factors (“Impact of distress”) pointed to changes in studying activities during the pandemic period. Students who found studying during the pandemic more difficult were also less motivated to study, did not use the extra time well, found the semester more stressful than previous ones, and had more difficulties adopting theoretical material for the exams due to the loss of practical courses. Hung et al. were among the first to report on the significant impact of the pandemic on difficulties with learning, particularly focusing on school work and difficulties in finding the motivation to study [28].

Students negatively appreciated the loss of practical courses, which is in concordance with the results of Iosif et al. and Hattar et al. [29,30]. Only around 16% of our students felt that the lost practical courses could be fully compensated, and clinical students were less likely to agree with that statement. While preclinical practical courses (i.e., practicals on manikins) are undoubtedly important in dental education for developing and training manual skills, the loss of clinical hours and practice on patients can hardly be replaced during dental education. Interestingly, in the present study, almost two-thirds of students reported that they would be willing to compensate for the lost clinical hours during their winter and summer holidays. However, only one-quarter would be willing to extend their studies, with preclinical students less willing to do so. Comparatively, Hung et al. reported a significantly higher reluctance to make up for the lost educational time and clinical experience, only up to 11% of the participants, in a population of undergraduate students and orthodontic residents through reduction of winter vacation, cancelling travel plans and the extension of working hours or the working week [28].

The impact of the suspension of clinical activities on long-term students’ professional competencies is not yet clear. Students themselves reported anticipating a decrease in their clinical professional skills, as highlighted in the studies by Loch et al. and Agius et al. [31,32]. Whether this may affect the quality of provided care can only be speculated.

The switch to distance learning could also be observed in terms of its social impact. A significant number of students reported missing the usual contact with the teachers and colleagues, 60% and 80%, respectively. Interestingly, one of the identified factors (“Modality of teaching”) suggests that students that were less likely to report to miss social contact with their teachers and peers, suggesting less need for social interaction, also appreciated various aspects of online learning more. With the rise of distance learning, a growing number of studies are looking to identify students’ personality traits and behaviours influencing their preferences for different modalities of education [33,34,35,36,37]. The data could be considered instrumental in structuring effective teaching and learning systems, accommodating different student learning styles.

It is important to highlight that e-learning has been used to various extents already employed in our school before the pandemic. Around 60% of students considered that the teaching faculty switched promptly to remote learning and organised the classes well in a short amount of time, and an even greater proportion agreed that the quality of classes improved over time. In another study by Puljak et al. conducted on a Croatian population of health science (non-dental) university students, the authors reported a similar percentage of agreement, 68%, with the efforts of their institutions to organise and adapt to online learning [38]. An important finding from our study in terms of possible improvements of online learning in the future is that over 90% of our students reported that the quality of classes differed significantly among different lecturers and different subjects. This may be an indicator of the need for further education of the teaching staff in the use of online tools.

A trend was also noticed concerning a factor related to “Satisfaction with online teaching”. Satisfied students rated the organisation of online classes and availability of lecturers for communication positively and also reported that online teaching improved over time, met their expectations, and gave them the same amount of information as they would expect to get in regular conditions.

Some of the frequently reported challenges of remote learning in the published literature are the technical problems and difficulties with access to technology [18,19,39,40,41]. This presents as another disparity between the developed and developing countries. For example, while almost all of our students had all the prerequisites to follow online classes, 37.5% of them encountered technical problems that made it impossible for them to follow classes at times. Due to the unprecedented increase in traffic, CARNet, provider of the online learning platform Merlin, faced difficulties in the first period upon switching to online learning. Shrivastava et al. reported that the most common problem encountered among dental students across India was internet connectivity [39]; conversely only 5% of students encountered this problem among the population of students in Giessen, Germany, as highlighted by Schlenz et al. [42].

This study presents some limitations. As it was conducted at only one dental school, the generalisation of the results is limited. Furthermore, at the inception of the study, no validated questionnaire was available; thus, we have created a new one. The full list of statements can be found in the tables accompanying this article.

Despite the gradual return of students to classrooms, we are witnessing a change in educational systems. A complete switch back to education exclusively in presence can hardly be expected. Thus, we believe that the data obtained by studies such as ours can improve students’ educational experience. As highlighted by our result, much of the responsibility for the quality and students’ appreciation of delivered online courses were directly related to lecturers. Thus, we have identified the need for further education of the faculty staff in the use of synchronous and asynchronous teaching tools. As practical classes are an integral part of the dental curriculum, their omission in the long perspective is unacceptable. As practical classes are re-assumed, a high level of protective measurements must be maintained to ensure safety, particularly for clinical practices. Overcrowding should be addressed by reorganising practical classes with smaller groups of students, in a manner and to the extent that institutional scheduling allows.

## 5. Conclusions

The COVID-19 pandemic affected a significant proportion of our student population negatively. While the students at our school reported relative satisfaction with changes implemented to their education due to the COVID-19 pandemic and introduction of online learning, this study has identified several challenges and obstacles that need to be addressed in the future. The results of this and similar studies may be used to implement changes in the existing online learning model, tailoring it better to students’ needs.

## Figures and Tables

**Table 1:** Sociodemographic characteristics of participants (N = 352).

Characteristic	N (%)
Age	22.5 ± 2.0
Gender	
Female	294 (83.5%)
Male	58 (16.5%)
**Year of study**	
First	46 (13.1%)
Second	67 (19.0%)
Third	53 (15.1%)
Fourth	63 (17.9%)
Fifth	71 (20.2%)
Sixth	52 (14.8%)
**Place of residence during lockdown**	
In the place of residence	323 (91.7%)
In Zagreb, which isn’t my place of residence	21 (6.0%)
Other	8 (2.3%)
**Living with during lockdown**	
Alone	12 (3.4%)
Family	311 (88.4%)
Partner	23 (6.5%)
Other	6 (1.7%)
**Tested on SARS-CoV-2**	
Yes, I was negative	21 (6.0%)
Yes, I was positive	6 (1.7%)
(I’ve experienced prejudice)	(5 (83.3%)
No, I haven’t been tested	325 (92.3%)

**Table 2:** The self-perceived psychoemotional impact of the COVID-19 pandemic.

Statement		Level of Agreement with the Statement					Mean ± SD		*p*-Value Gender	*p*-Value Pre/Clinical
		1	2	3	4	5				
S1	The emergence of the SARS-CoV-2 made me feel concerned and insecure.	13.1%	24.1%	31.5%	22.2%	9.1%	Total	2.9 ± 1.2	0.205	0.819
							Female	2.9 ± 1.2		
							Male	2.7 ± 1.1		
							Preclinical	2.9 ± 1.2		
							Clinical	2.9 ± 1.2		
S2	The introduction of a strict public health measure, i.e., lockdown, in Croatia made me feel concerned and insecure.	2.0%	10.8%	21.0%	45.7%	20.5%	Total	3.7 ± 1.0	0.043 *	0.490
							Female	3.8 ± 1.0		
							Male	3.5 ± 1.0		
							Preclinical	3.7 ± 1.0		
							Clinical	3.8 ± 1.0		
S3	I feel fear and concern about the possibility of SARS-CoV-2 contagion.	11.4%	28.7%	33.5%	21.9%	4.5%	Total	2.8 ± 1.1	0.128	0.688
							Female	2.8 ± 1.1		
							Male	2.6 ± 1.0		
							Preclinical	2.8 ± 1.1		
							Clinical	2.8 ± 1.0		

* Differences between genders and between preclinical and clinical students were tested using a two-tailed *t*-test. Significant difference (*p* < 0.05); 1—completely disagree, 2—disagree, 3—neither agree, nor disagree, 4—agree, 5—completely agree.

**Table 3:** The self-perceived educational impact of the COVID-19 pandemic.

Statement		Level of Agreement with the Statement					Mean ± SD		*p*-Value Gender	*p*-Value Pre/Clinical
		1	2	3	4	5				
S4	I was concerned about the outcome of the academic year 2019/20.	7.7%	14.5%	15.1%	32.7%	30.1%	Total	3.6 ± 1.3	<0.001 *	0.780
							Female	3.8 ± 1.2		
							Male	2.9 ± 1.3		
							Preclinical	3.7 ± 1.3		
							Clinical	3.6 ± 1.2		
S5	The summer semester of the academic year 2019/20 was more stressful than the previous ones.	13.4%	24.1%	23.6%	18.8%	20.2%	Total	3.1 ± 1.3	<0.001 *	0.323
							Female	3.2 ± 1.3		
							Male	2.4 ± 1.1		
							Preclinical	3.2 ± 1.3		
							Clinical	3.0 ± 1.4		
S6	Due to the uncertainty caused by the COVID-19 pandemic, I was not equally motivated to study.	17.3%	22.7%	22.2%	18.5%	19.3%	Total	3.0 ± 1.4	0.038 *	0.014 *
							Female	3.1 ± 1.4		
							Male	2.7 ± 1.3		
							Preclinical	3.2 ± 1.4		
							Clinical	2.8 ± 1.4		
S7	During the period of strict public health measures (i.e., lockdown) studying was more difficult than usual.	19.0%	21.9%	16.8%	24.4%	17.9%	Total	3.0 ± 1.4	0.005 *	0.002 *
							Female	3.1 ± 1.4		
							Male	2.5 ± 1.3		
							Preclinical	3.2 ± 1.3		
							Clinical	2.8 ± 1.4		
S8	During the period of strict public health measures (i.e., lockdown) I had more time for studying, which I’ve used well and have benefited from.	8.2%	21.0%	23.6%	28.1%	19.0%	Total	3.3 ± 1.2	0.055	0.355
							Female	3.2 ± 1.2		
							Male	3.6 ± 1.2		
							Preclinical	3.2 ± 1.3		
							Clinical	3.3 ± 1.2		
S9	I missed social contact with teachers.	5.7%	9.4%	24.7%	31.0%	29.3%	Total	3.7 ± 1.2	0.064	0.010 *
							Female	3.7 ± 1.1		
							Male	3.4 ± 1.3		
							Preclinical	3.9 ± 1.1		
							Clinical	3.5 ± 1.2		
S10	I missed social contact with colleagues.	2.8%	2.8%	4.0%	24.1%	66.2%	Total	4.5 ± 1.0	0.548	0.058
							Female	4.5 ± 0.9		
							Male	4.4 ± 1.0		
							Preclinical	4.6 ± 0.8		
							Clinical	4.4 ± 1.0		
S11	Adaptation in knowledge assessment/examination (online exams) was a source of stress for me.	18.8%	24.4%	19.0%	24.7%	13.1%	Total	2.9 ± 1.3	0.034 *	0.071
							Female	3.0 ± 1.3		
							Male	2.6 ± 1.3		
							Preclinical	3.0 ± 1.2		
							Clinical	2.8 ± 1.4		
S12	Adaptation in knowledge assessment/examination (oral exams in-person, with PPE—gloves and face masks) was a source of stress for me.	21.9%	29.5%	23.9%	16.2%	8.5%	Total	2.6 ± 1.2	0.050	0.462
							Female	2.7 ± 1.3		
							Male	2.3 ± 1.1		
							Preclinical	2.7 ± 1.2		
							Clinical	2.6 ± 1.3		
S13	The teachers were as available for contact and communication as usual.	5.4%	17.0%	27.6%	34.1%	15.9%	Total	3.4 ± 1.1	0.523	0.326
							Female	3.4 ± 1.1		
							Male	3.5 ± 1.0		
							Preclinical	3.3 ± 1.2		
							Clinical	3.4 ± 1.0		
S14	The suspension and loss of practical courses in the summer semester of the academic year 2019/20 can be fully compensated.	36.6%	28.4%	18.5%	8.8%	7.7%	Total	2.2 ± 1.3	0.490	<0.001 *
							Female	2.2 ± 1.3		
							Male	2.3 ±1.2		
							Preclinical	2.5 ± 1.2		
							Clinical	2.0 ± 1.2		
S15	The suspension and loss of practical courses affected my knowledge during knowledge assessments (exams).	7.7%	18.5%	28.1%	26.4%	19.3%	Total	3.3 ± 1.2	0.146	0.185
							Female	3.4 ± 1.2		
							Male	3.1 ± 1.1		
							Preclinical	3.2 ± 1.2		
							Clinical	3.4 ± 1.2		
S16	Due to the suspension and loss of practical courses, it was more difficult to understand and adopt the study materials needed for the knowledge assessments (exams).	7.7%	19.0%	26.1%	26.4%	20.7%	Total	3.3 ± 1.2	0.266	0.684
							Female	3.4 ± 1.2		
							Male	3.2 ± 1.1		
							Preclinical	3.3 ± 1.2		
							Clinical	3.4 ± 1.2		
S17	If there was an opportunity to compensate for the lost practical courses during the summer and/or winter holidays, I would agree to that.	7.4%	11.9%	21.9%	29.5%	29.3%	Total	3.6 ± 1.2	0.514	<0.001 *
							Female	3.6 ± 1.2		
							Male	3.5 ± 1.2		
							Preclinical	3.3 ± 1.2		
							Clinical	3.9 ± 1.2		
S18	If there was an opportunity to compensate for the lost practical courses by extending my graduate studies, I would agree to that.	35.2%	19.6%	19.6%	12.2%	13.4%	Total	2.5 ± 1.4	0.250	<0.001 *
							Female	2.5 ± 1.5		
							Male	2.3 ± 1.2		
							Preclinical	2.1 ± 1.1		
							Clinical	2.8 ± 1.5		

* Differences between genders and between preclinical and clinical students were tested using a two-tailed *t*-test. Significant difference (*p* < 0.05); 1—completely disagree, 2—disagree, 3—neither agree, nor disagree, 4—agree, 5—completely agree.

**Table 4:** Students’ attitudes and perception of online learning.

Statement		Level of Agreement with the Statement					Mean ± SD		*p*-Value Gender	*p*-Value Pre/Clinical
		1	2	3	4	5				
S19	I had all the prerequisites enabling me to follow online classes	0.6%	3.1%	4.5%	23.6%	68.2%	Total	4.4 ± 0.9	0.382	0.244
							Female	4.5 ± 0.8		
							Male	4.6 ± 0.6		
							Preclinical	4.5 ± 0.8		
							Clinical	4.6 ± 0.7		
S20	I encountered technical issues that sometimes made it impossible to follow online classes.	26.4%	21.6%	14.5%	26.4%	11.1%	Total	2.8 ± 1.4	0.407	0.251
							Female	2.8 ± 1.4		
							Male	2.6 ± 1.3		
							Preclinical	2.8 ± 1.4		
							Clinical	2.7 ± 1.4		
S21	During online classes, I received the same amount of information as did the previous student generations in the same period.	15.9%	23.3%	25.6%	23.9%	11.4%	Total	2.9 ± 1.3	0.039 *	0.450
							Female	2.9 ± 1.3		
							Male	3.2 ± 1.2		
							Preclinical	2.9 ± 1.2		
							Clinical	3.0 ± 1.3		
S22	Online lectures are as valuable as onsite lectures.	9.4%	13.6%	15.1%	22.4%	39.5%	Total	3.7 ± 1.4	0.600	<0.001 *
							Female	3.7 ± 1.4		
							Male	3.8 ± 1.3		
							Preclinical	3.4 ± 1.4		
							Clinical	3.9 ± 1.3		
S23	I was able to be more focused during online lectures than I would during onsite lectures.	12.8%	11.1%	18.5%	20.2%	37.5%	Total	3.6 ± 1.4	0.680	0.008 *
							Female	3.6 ± 1.4		
							Male	3.7 ± 1.2		
							Preclinical	3.4 ± 1.4		
							Clinical	3.8 ± 1.4		
S24	I would master the curriculum more successfully if in person/direct contact with the teacher.	10.5%	21.0%	34.1%	17.6%	16.7%	Total	3.1 ± 1.2	0.571	0.022 *
							Female	3.1 ± 1.2		
							Male	3.2 ± 1.3		
							Preclinical	3.3 ± 1.1		
							Clinical	3.0 ± 1.3		
S25	Lectures and theoretical courses could be carried out online (virtually) in the future as well.	6.8%	8.8%	14.8%	19.0%	50.6%	Total	4.0 ± 1.3	0.294	0.268
							Female	3.9 ± 1.3		
							Male	4.1 ± 1.2		
							Preclinical	3.9 ± 1.2		
							Clinical	4.0 ± 1.3		
S26	The use of online education platforms prepared me well for further education and improvement.	5.1%	14.2%	36.4%	25.6%	18.8%	Total	3.4 ± 1.1	0.639	0.040 *
							Female	3.4 ± 1.1		
							Male	3.4 ± 0.9		
							Preclinical	3.3 ± 1.1		
							Clinical	3.5 ± 1.1		
S27	The teaching faculty managed to organise online classes well in the short time frame.	6.3%	11.6%	23.6%	35.2%	23.3%	Total	3.6 ± 1.2	0.750	0.186
							Female	3.6 ± 1.2		
							Male	3.6 ± 1.0		
							Preclinical	3.7 ± 1.2		
							Clinical	3.5 ± 1.1		
S28	The quality of online classes differed among subjects.	0.6%	2.0%	6.8%	26.4%	64.2%	Total	4.5 ± 0.8	0.089	0.071
							Female	4.5 ± 0.8		
							Male	4.4 ± 0.7		
							Preclinical	4.4 ± 0.8		
							Clinical	4.6 ± 0.7		
S29	The quality of online classes differed among the members of the teaching faculty.	0.6%	1.1%	2.8%	26.7%	68.8%	Total	4.6 ± 0.7	0.083	0.004 *
							Female	4.6 ± 0.6		
							Male	4.5 ± 0.7		
							Preclinical	4.5 ± 0.7		
							Clinical	4.7 ± 0.6		
S30	The quality of online classes improved over time (over the course of the semester).	4.3%	6.5%	27.8%	40.3%	21.0%	Total	3.7 ± 1.1	0.677	0.734
							Female	3.7 ± 1.1		
							Male	3.7 ± 0.8		
							Preclinical	3.7 ± 1.0		
							Clinical	3.7 ± 1.0		
S31	Online teaching influenced the amount of the acquired knowledge.	2.0%	6.0%	27.6%	38.6%	25.9%	Total	3.8 ± 1.0	0.148	0.472
							Female	3.8 ± 1.0		
							Male	3.6 ± 0.9		
							Preclinical	3.8 ± 0.9		
							Clinical	3.8 ± 1.0		
S32	Online teaching influenced the results of the exams I sat.	4.0%	16.5%	27.0%	31.0%	21.6%	Total	3.5 ± 1.1	0.076	0.199
							Female	3.5 ± 1.1		
							Male	3.3 ± 1.1		
							Preclinical	3.6 ± 1.0		
							Clinical	3.4 ± 1.2		
S33	Online teaching fully met my expectations.	10.8%	16.5%	36.6%	25.0%	11.1%	Total	3.1 ± 1.1	0.062	0.918
							Female	3.0 ± 1.2		
							Male	3.3 ± 1.0		
							Preclinical	3.1 ± 1.2		
							Clinical	3.1 ± 1.1		

* Differences between genders and between preclinical and clinical students were tested using a two-tailed *t*-test. Significant difference (*p* < 0.05); 1—completely disagree, 2—disagree, 3—neither agree, nor disagree, 4—agree, 5—completely agree.

**Table 5:** Perceptions and attitudes toward SARS-CoV-2, teaching, and online teaching during COVID-19 pandemic grouped in dimensions by explanatory factor analysis.

Factor		Statements	Loading
1. Modality of teaching	S25	Lectures and theoretical courses could be carried out online (virtually) in the future as well.	0.794
	S23	I was able to be more focused during online lectures than I would during onsite lectures.	0.776
	**S24**	**I would master the curriculum more successfully if in person/direct contact with the teacher.**	**−0.763**
	S22	Online lectures are as valuable as onsite lectures.	0.732
	S26	The use of online education platforms prepared me well for further education and improvement.	0.625
	**S9**	**I missed social contact with teachers.**	**−0.556**
	**S10**	**I missed social contact with colleagues.**	**−0.434**
2. Impact of distress	S7	During the period of strict public health measures (i.e., lockdown), studying was more difficult than usual.	0.862
	S6	Due to the uncertainty caused by the COVID-19 pandemic, I was not equally motivated to study.	0.827
	**S8**	**During the period of strict public health measures, I had more time for studying, which I’ve used well and have benefited from.**	**−0.685**
	S5	The summer semester of the academic year 2019/20 was more stressful than the previous ones.	0.522
	S16	Due to the suspension and loss of practical courses, it was more difficult to understand and adopt the study materials needed for the exams.	0.449
3. Satisfaction with online teaching	S27	The teaching faculty managed to organise online classes well in the short time frame.	0.814
	S13	The teachers were as available for contact and communication as usual.	0.698
	S33	Online teaching fully met my expectations.	0.656
	S30	The quality of online classes improved over time (over the course of the semester).	0.630
	S21	During online classes, I received the same amount of information as did the previous generations in the same period.	0.457
4. Response to the pandemic	S3	I was and am feeling fear and concern about the possibility of becoming infected with SARS–CoV–2.	0.752
	S2	The introduction of a strict public health measure, i.e., lockdown, in Croatia made me feel concerned and insecure.	0.748
	S1	The emergence of the SARS-CoV-2 made me feel concerned and insecure.	0.743
	S4	I was feeling concerned about the outcome of the academic year 2019/20.	0.422

Positive correlation coefficients, **negative correlation coefficients**.

## Data Availability

Not applicable.
